# Impacts of new cycle infrastructure on cycling levels in two French cities: an interrupted time series analysis

**DOI:** 10.1186/s12966-022-01313-0

**Published:** 2022-07-07

**Authors:** Christina S. Xiao, Stephen J. Sharp, Esther M. F. van Sluijs, David Ogilvie, Jenna Panter

**Affiliations:** MRC Epidemiology Unit, School of Clinical Medicine, University of Cambridge, Cambridge, UK

**Keywords:** Cycle lane infrastructure, Urban health, Transport policy, Interrupted time series analysis

## Abstract

**Background:**

Cities globally have started to make substantial investment in more sustainable forms of transportation. We aimed to evaluate whether the construction of new cycling infrastructure in Paris and Lyon, France, affected population cycling activity along new or improved routes.

**Methods:**

Routinely collected cycle count data from January 2014 to March 2020 were acquired for the cities of Paris and Lyon. Improvements were identified at 15 locations with 6 months of pre- and post-intervention data. Comparison streets were chosen within Paris or Lyon for which pre-intervention trends in cycling were similar to those at intervention sites. Controlled interrupted time series analyses and autocorrelation were performed adjusting for seasonality. Random-effects meta-analysis combined results across streets within each city and overall.

**Results:**

On average, cycling counts/day increased on both intervention and control streets in Paris and Lyon. In general, results of the ITS analysis indicated no significant change in the level or trend as a result of the improvements in either city. Meta-analysis suggested that intervention streets in Paris had a larger positive pooled effect size for level change (218 cycle counts, 95% CI -189, 626, I^2^ = 0%) compared to Lyon (34, 95% CI -65, 133, I^2^ = 14%); however, confidence intervals for both cities were wide and included no effect.

**Conclusions:**

The findings suggest that improving or constructing new cycle lanes may be necessary but not sufficient to induce significant changes in cycling levels. There is a need to understand how context, intervention design and other complementary interventions can improve the effectiveness of new cycling infrastructure.

**Supplementary Information:**

The online version contains supplementary material available at 10.1186/s12966-022-01313-0.

## Background

With almost 70% of the world population predicted to live in urban environments by 2050 [1, 2], how we design our cities is increasingly important. Across the globe, many cities have historically been designed to prioritise car use rather than more active and sustainable modes of transportation, such as walking and cycling [3, 4]. Motor vehicle use is not only a significant source of carbon emissions, which contribute to worsened air quality and climate change, but it also reduces opportunities for physical activity. Inactivity and poor air quality have been associated with increased risk of mortality and various chronic diseases, such as cardiovascular disease, respiratory illness, obesity, several types of cancers, and depression [5, 6].

Transport policies that encourage walking and cycling instead of driving offer opportunities to tackle these pressing health and environmental challenges. One strategy for reducing car dependency and promoting active travel is constructing or improving cycle lanes. Systematic reviews of natural experimental studies have found that these interventions have been effective in increasing levels of cycling [7–9]. However, many studies were found to be of low quality or have a high risk of bias, with few studies having control areas or streets and limited adjustment for potential confounders [7, 8, 10]. Studies also tended to use repeat cross-sectional designs with two time points to assess change, meaning the analyses could not account for baseline trends [7, 9, 10].

More robust analytical approaches such as interrupted time series (ITS) analysis have been rarely used to evaluate new cycling infrastructure [11], perhaps due to the large number of time points needed. Strengths of controlled ITS include that it makes full use of the data, accounts for differences in underlying baseline trends, and takes into account other potential confounders such as co-interventions or contemporaneous events [12–14]. It has been widely used in public health research to examine a large variety of interventions, such as cycle helmet laws, infectious disease control, and public health mass media campaigns such as air quality alert programs [15–18].

There are growing opportunities to evaluate the impacts of new infrastructure with continuous monitoring data collected in real time from automatic counters, remote-sensing mobile applications and the like, particularly where these are publicly available [19]. For example, in France, cities such as Paris and Lyon have monitored cycling levels on streets across the city and recently prioritized improving their cycling infrastructure as part of their transportation strategies. Using a controlled ITS analysis, we evaluate whether and how the construction of different individual segments of cycling infrastructure introduced between 2014 and 2019 affected cycling levels in Paris and Lyon.

## Methods

### Intervention

Many cities in France have invested in their cycling networks to promote active travel and reduce vehicular emissions. Among the most ambitious are Paris, the capital and most densely populated city (20,300 inhabitants per square kilometre), and Lyon, the third most densely populated city (10,000 inhabitants per square kilometre). Both cities have similar climates, generally flat terrains and relatively well-connected public transport networks, with low cycling mode shares of approximately 3% before interventions were introduced [20, 21]. Each city also included targets such as increasing the number of traffic-calmed streets by introducing 30 km (km)/hour zones or adding cycle parking; they also included subsidies for cargo or electric bikes [22–24]. However, there are several differences between the two cities’ respective plans involving other policies that may influence cycling levels. Paris implemented annual and weekly car-free days (Journée Sans Voiture and Paris Respire) in certain neighbourhoods and a series of low emission zones which have restricted older diesel vehicles from entering the city [25–27].

Each city’s cycling plans involved expanding the length of its cycle lanes (from 730 to 1400 km in Paris and from 520 to 920 km in Lyon) [22, 24]. This study evaluates changes made to the 18 streets for which before and after cycle count data are available. Table 1 describes the infrastructural changes made to each intervention street included in this study. Cycle lanes were lengthened by between 0.1 and 2.0 km. In most cases in both cities, the space dedicated to cyclists was widened (13/18 streets). Paris mostly introduced physically separated lanes (5/8 streets) and allowed cyclists to go both with and against traffic (i.e., bidirectionally). In addition to constructing physically separated lanes (3/10 streets), the city of Lyon installed more diverse infrastructure (3/10 streets), which included adding shared lane markings (2/10 streets), installing painted cycle lanes (3/10 streets), and converting painted cycle lanes to shared bus lanes (2/10).

**Table 1:** Changes in cycle lane features in intervention streets

Intervention street	Increase in length (km)	Width	Infrastructure type ^a^	Direction ^b^
**Paris**				
Rue de Rivoli	1.7	Increase	Added physically segregated cycle lane	Added bi-directionality
Boulevard Voltaire	1.4	Increase	Added physically segregated cycle lane	Added bi-directionality
Rue Julia Bartet*	0.3	No change	No change	No change
Boulevard Diderot	0.1	Increase	Added painted cycle lane	No change
Rue d'Aubervilliers	0.3	Increase	No change	Added bi-directionality
Avenue de la Porte des Ternes	0.2	No change	Added physically segregated cycle lane	No change
Rue de Turbigo	0.2	Increase	Added physically segregated cycle lane	Added bi-directionality
Rue Lecourbe	0.6	Increase	Added physically segregated cycle lane	Added bi-directionality
**Lyon**				
Rue Victor Lagrange	0.5	Increase	Added shared lane marking (sharrow)	Added contra-traffic direction
Quai Hippolyte Jaÿr	0.2	Increase	Added physically segregated cycle lane	Added bi-directionality
Cours Gambetta	0.4	Decrease	Converted cycle lane to shared bus lane	No change
Quai Claude Bernard	0.6	Increase	Added physically segregated cycle lane	Added bi-directionality
Rue de la Viabert	0.1	Increase	Painted cycle lane	Added bi-directionality
Cours Albert Thomas	1.5	Decrease	Converted cycle lane to shared bus lane	No change
Avenue Félix Faure	0.1	Increase	Added painted cycle lane	Added bi-directionality
Rue Vauban	0.2	No change	Added painted cycle lane	Added contra-flow direction
Rue Rabelais	0.2	Increase	Added shared lane marking (sharrow)	Added contra-flow direction
Boulevard Pinel	2.0	Increase	Added physically segregated cycle lane	Added bi-directionality

^*^ The only change was a new surface treatment of the existing cycle lane

^a^ Sharrows comprise a sign of a bicycle with or without wide arrows painted on road surfaces to indicate where cyclists and motorists should share the road

^b^ Bi-directional cycle lanes refer to cycle lanes which allow cyclists to go with and against traffic on the same side of the road; contra-flow cycle lanes refer to those which allow cyclists to travel against traffic

### Data collection

We acquired routinely collected cycle count data from approximately 110 cycle counters (40 in Paris and 74 in Lyon) between January 2014 and March 2020 from an open data repository for Paris [28], or by web-scraping Eco Counter, an online cycle count application for Lyon [29]. Web-scraping involved using Python to extract and export data hosted on the application into a structured format such as a table. These repositories also included datasets on which streets received cycle lane improvements, a brief description of what improvements were made and when they were delivered. When we could not identify information using these methods, we contacted representatives from the city transport authorities by email.

We identified 18 newly built cycle infrastructure improvements from the datasets with cycle count data before the interventions were implemented. Periods of pre-intervention and potential follow-up time differed, as the installation of cycle counters and construction or renovation of cycle lanes did not appear to be systematic. Furthermore, the coronavirus pandemic shortened the follow-up date to March 16, 2020, the date that France entered confinement due to the coronavirus [30]. We did not include travel patterns after this time point because they were not representative of typical behaviour.

### Outcome measure

The outcome was daily cycle counts as measured by automatic cycle counters distributed throughout Paris and Lyon. Both cities used ZELT, or permanent cycle counters embedded in the ground which measured counts by detecting the metal in bicycle wheels using induction loop technology (Personal communication, 14 September 2021). These types of cycle counters have been used in other studies, which have reported an accuracy of 94% to 98% in differentiating bikes from other forms of transport such as cars, motorcycles, and larger vehicles [31, 32]. In Lyon, daily counts were presented and therefore for comparability, data for Paris were aggregated from hourly counts to daily counts.

### Control streets

**Fig. 1:**
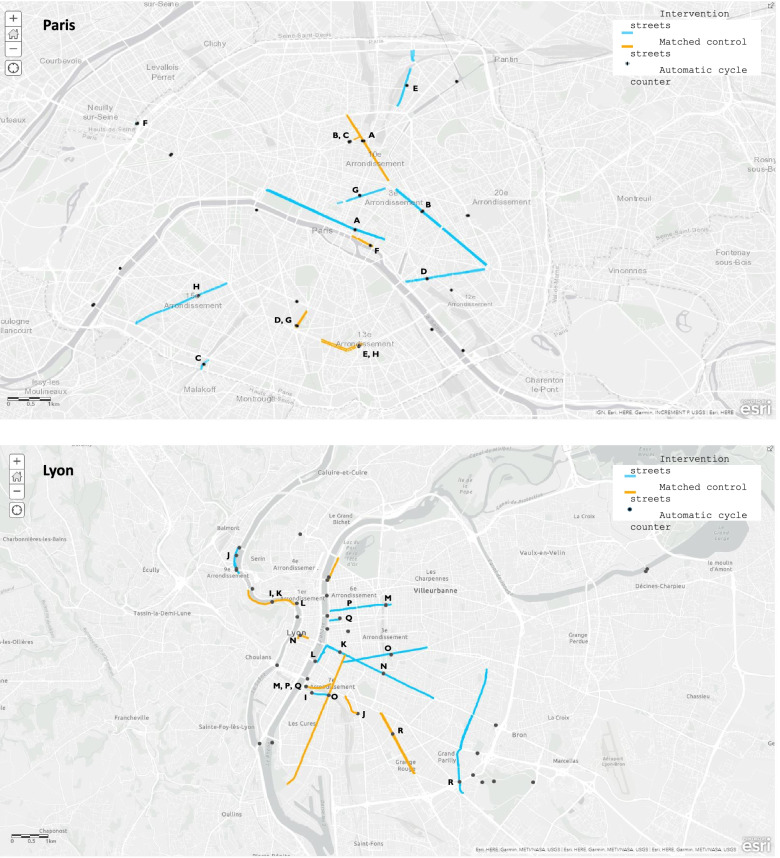
Location of intervention and control streets in Paris and Lyon*

Because there were multiple control streets available to choose from for every intervention street, several steps were followed to select control streets. First, potential control streets were selected which had the same 6 month pre- and post-intervention time-period as the intervention street to increase comparability and to ensure all streets shared the same amount of pre-and post-period data. We then plotted pre-intervention trends for each intervention and control street; potential control streets with parallel pre-intervention trends and similar levels to the intervention street were selected. Potential control street cycle counters were mapped to verify they were not in close proximity (< 2 km) to intervention street cycle counters to avoid capturing any potential contamination or displacement (Fig. 1). Lastly, pre-intervention street features (e.g., number of traffic lanes, cycling infrastructure) were compared between potential control and intervention streets using Google Street View to make a definitive selection of control streets. For instance, control streets were selected if their pre-intervention infrastructure was of the same type (e.g., painted cycle lane, shared bus lane, or no cycle lane infrastructure) as those on intervention streets before changes were made. Appendix Table 1 lists intervention streets with their selected control streets.

**Fig. 2:**
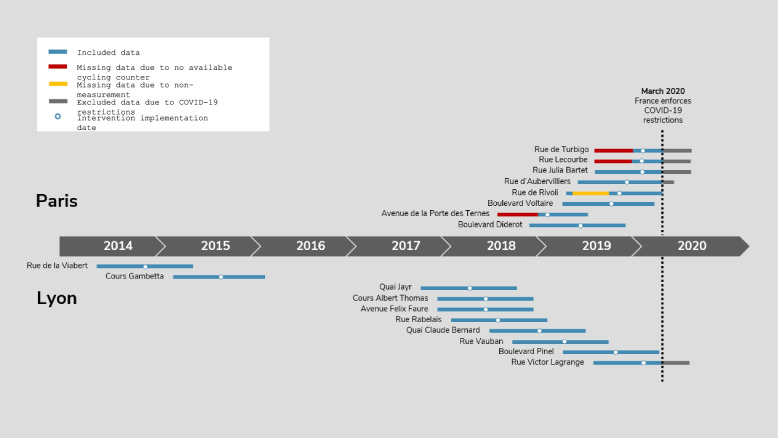
Study period timeline for intervention streets in Paris and Lyon (refer to Appendix Table 2 for exact dates)

Figure 2 and Appendix Table 2 describe the data assessment periods for each street in Paris and Lyon before and after intervention implementation. It should be noted that several intervention streets, specifically Rue Julia Bartet and Rue d’Aubervilliers in Paris and Rue Victor Lagrange in Lyon, did not have a full six months of follow-up data available due to the coronavirus lockdown restrictions. In addition, a significant portion of the pre-intervention data was missing for Rue de Rivoli, possibly due to the removal of the cycle counter when installing the new cycling infrastructure. For Rue de Turbigo, Rue Lecourbe, and Avenue de la Porte des Ternes, cycle counters were installed less than one month before intervention implementation. We excluded these three streets from the primary analysis due to the shortened pre-intervention period; their results are presented in Appendix Table 3. This left 15 intervention streets that were evaluated in the main analysis.

### Interrupted time series analysis

For each pair of intervention and control streets, a segmented linear regression model was used to estimate the effect of the intervention on the level change (change in level between time points immediately before and after the intervention accounting for the pre-intervention trend) and the change in trend (difference between pre-intervention and post-intervention slopes) for cycling counts [12]. Autocorrelation between residuals was assessed using a partial autocorrelation plot and the Durbin-Watson statistic and incorporated using an autoregressive integrated moving average (ARIMA) model with the nlme package in R [33]. Fourier terms were included in the model to adjust for seasonality using the R package tsModel [34, 35]. Appendix page 4 describes the regression model in full. For Paris, additional parameters were included in the model to account for a transportation strike between December 5, 2019 and January 27, 2020 [36], as this may have impacted cycling levels [37, 38].

### Meta-analysis

To assess the impact of the intervention on cycling level and trend, we performed a random-effects meta-analysis using the R package **meta** to pool estimates of the intervention effect from the different pairs of streets within each city and then across both cities [39]. We also calculated the I^2^ statistic, the percentage of the variability in effect estimates due to heterogeneity. We conducted all analyses in R (version 4.0.4) [40].

### Sensitivity analyses

We performed three sets of sensitivity analyses. The first assessed whether changing the control street selection affected the results. The mean of the values from all potential control streets at each time point was calculated and was used to reflect general city-wide trends. We also examined whether choosing streets which shared the same time-period of data collection as the intervention streets may have changed results, to remove any possible effect of other events which may have occurred during that period. For the second sensitivity analysis, where data were available, the assessment period was extended up to one year both in the pre-and post-intervention period to determine whether intervention effects were present in the longer term. The third analysis introduced a one-month lag effect to assess whether introducing the cycling infrastructure has effects one month after implementation compared to immediate effects at intervention implementation.

## Results

### Mean changes in cycle counts

**Fig. 3:**
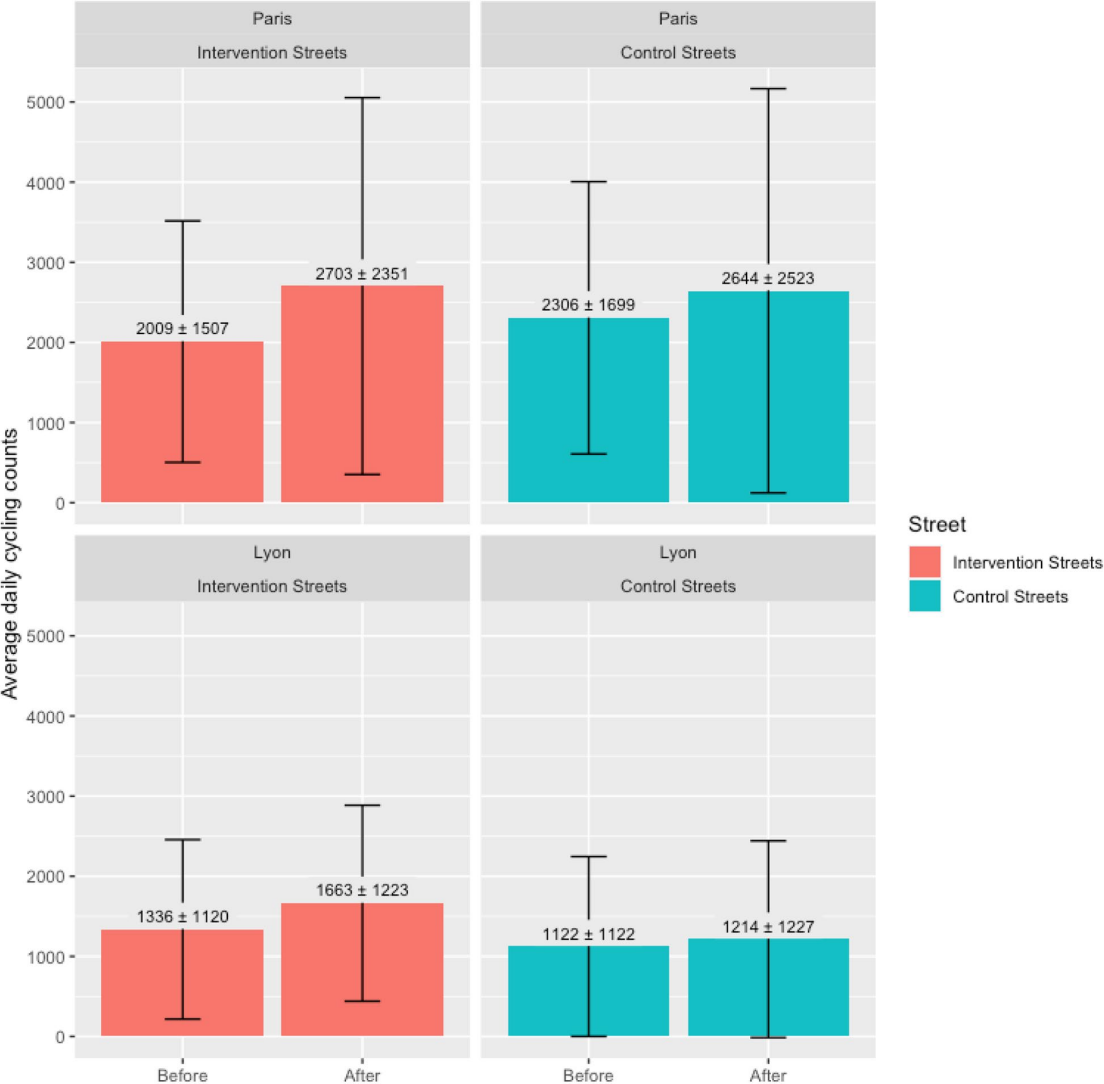
Before and after mean daily cycling counts on intervention and control streets in Paris and Lyon with standard deviations denoted by the error bars

Across all intervention and control streets, a mean of 1864 cyclists were counted per day during the study period (Fig. 3). Mean daily cycling counts on intervention streets in Paris increased by 694 (34.5%), while a smaller increase of 338 cyclists (14.7%) was observed on their control streets over the same time period. On intervention streets in Lyon, mean daily cycling counts increased by 327 (24.5%). Mean daily cycling counts also increased by a smaller amount on control streets in Lyon, with an increase of 92 (8.2%).

### Interrupted time series results

**Fig. 4:**
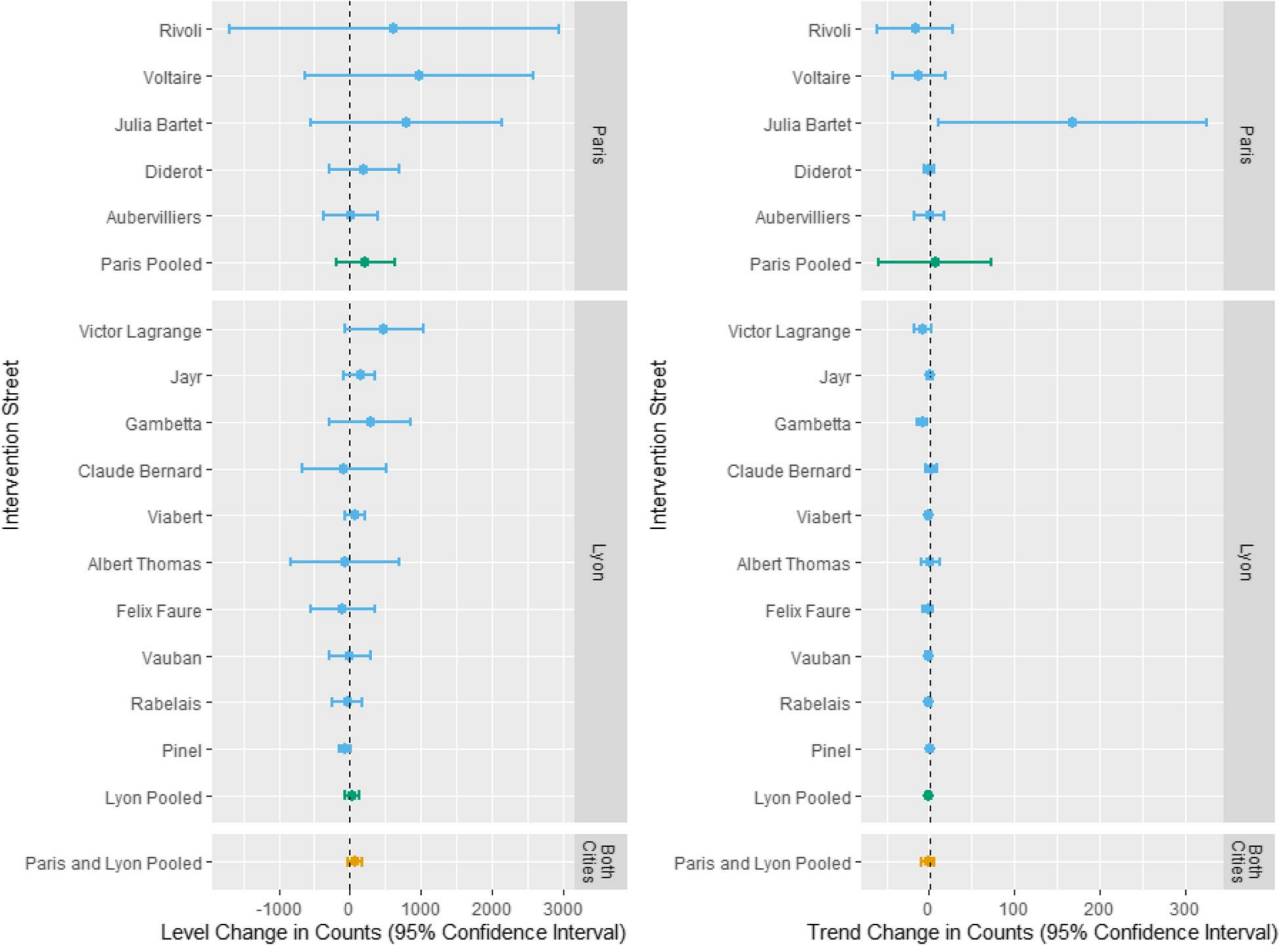
Estimated effects of intervention for each individual street and combined across streets

Although there were positive changes in cycling counts on some streets (e.g., 982/day on Boulevard Voltaire), in general, we did not detect an effect of new cycling infrastructure immediately after this was introduced compared to control streets, as shown by the confidence intervals crossing 0 (Fig. 4 and Appendix Table 4). Changes in trend were small and largely non-statistically significant. However, there were a few exceptions: there was a large increase of 168 counts/day (95% CI 11, 325) on Rue Julia Bartet and a decrease of -7 counts/day (95% CI -14, -2) on Cours Gambetta.

The meta-analysis showed that streets in Paris had a greater positive pooled effect size for the level change (218 counts, 95% CI -189, 626, I^2^ = 0%) than Lyon (34, 95% CI -65, 133; I^2^ = 14%), although confidence intervals for both cities were wide. The pooled effect size across both cities for changes in level for all streets was small but positive (68, 95% CI -36, 172; I^2^ = 3%). There were no noticeable changes in pooled cycling count trends for intervention streets compared to control streets, either with each city or across both cities. Appendix Fig. 1 displays an example of an ITS figure for Boulevard Voltaire, where the effects of the public transport strike can be clearly seen.

### Sensitivity analyses results

There were no substantial differences in the ITS results using different control groups, extending the study period, or introducing an intervention lag effect of one month (Appendix Fig. 2). There were a few exceptions; for instance, with the comparison group being an average of all control streets, on Boulevard Pinel in Lyon, there was a significant decrease of -313 counts per day (95% CI: -622, -3). Introducing a one-month lag for Rue Gambetta changed the results from a significant decrease to a significant increase in trend of 4 (95% CI 0, 8) (Appendix Fig. 3). After introducing a one-month lag, previously statistically significant results for the level (Rue de Rivoli) and trend (Rue Julia Bartet) were no longer significant.

## Discussion

We evaluated the impact of 15 new or improved cycle lanes in Paris and Lyon on cycling using a controlled ITS analysis. Cycle counts increased on both intervention and control streets in Paris and Lyon. Furthermore, there were positive yet non-statistically significant changes on individual streets immediately after (as indicated by the change in level) or six months after the improvements (as indicated by the changes in trend), except on Rue Julia Bartet. As the changes on this street were introduced during the public transport strike, some of the trend changes may be due to more people switching from public transport to cycling as the strike continued. Altogether, however, these findings suggest that improving or constructing new cycle lanes may be necessary but not sufficient to induce significant changes in cycling levels.

We also found that meta-analysis pooled point estimates for changes in cycling counts were larger in Paris than Lyon, which may be attributable to differences in contexts and additional policies introduced in Paris during the study period, such as the introduction of more ‘stick’ interventions (e.g., ban on diesel vehicles). Indeed, some research has suggested that a combination of carrot and stick interventions, which include both positive and negative motivators for active travel, respectively, may be more effective than either alone [41, 42]. Furthermore, the design of the cycle lanes may have been more supportive of increasing cycling levels in Paris than Lyon. Compared to Lyon, there were more cycle lanes separated from motor traffic introduced in Paris, which certain groups of cyclists (e.g., women) have been found to prefer [43, 44]. However, the effects of these differences were not formally assessed, being beyond the scope of the study.

Previous studies using primarily before-and-after study designs have found that increasing the quantity of new or improved cycle infrastructure was associated with increases in cycling behaviour [45–47], only one of which has used an ITS analysis, albeit with an uncontrolled study design [11]. Consistent with the findings of our study, other studies that included a comparison group found either no or unclear effects of improved cycling infrastructure [48, 49]. Some systematic reviews have observed that studies with weaker study designs were more likely to report significant changes in active travel outcomes than those with more robust analytical methods [9]. This is in line with the non-significant findings of our study, which uses a controlled study design with adjustment for baseline trends through ITS analysis.

There may be other reasons we did not find this improved cycle infrastructure to have a statistically significant effect. For example, cycle lanes may not immediately affect cycling levels or may take more than six months to be effective. Other studies have reported a significant change in walking and cycling levels in the longer term [9], including two years after introducing new infrastructure [50]. Furthermore, one systematic review of the effects of cycling infrastructural changes on physical activity found that studies which had an exposure period of fewer than six months were more likely to find that interventions were not effective [7]. The national Covid-19 lockdown in March 2020 greatly affected cycling levels throughout both cities, and this study cannot therefore provide evidence for effects beyond 6 months after infrastructure construction. However, we did test the effect of extending the length of follow-up for as long as possible before the lockdown, and the results of this sensitivity analysis did not differ substantially from those of the main analyses. Future work should explore longer term effects where possible.

Similarly, another possible explanation for our results is that cycling infrastructure, particularly those involving constructing new lanes, may not be introduced all at once. For longer streets, improvements may have been delivered in several stages. We used the date listed in the databases as the final cut-off point for implementation, as further information about the exact date the other stages were delivered was not available. This may also explain why we did not see significant changes in either the level or trend; it may be that the true effect of the intervention accumulates with each successive phase of construction. Further studies assessing cycle lanes may want to take this into account, and if they have the necessary information on when these were implemented, should use the appropriate methods which take into account these additional interventions [51].

The selection of control streets within the same city may have attenuated any detectable differences since both intervention and control streets are part of the same cycling network. Strengthening a given section of the cycling network may improve the whole network of which the control streets form part, particularly if control streets are within cycling distance of intervention streets (i.e., a contamination or spill-over effect). For instance, increasing the connectivity of the cycling network may encourage cyclists to use control streets to reach the improved intervention streets. Indeed, cycling levels in both Paris and Lyon have been increasing since the early 2000’s [52, 53]. We attempted to reduce the possibility of contamination effects by choosing control streets that were more than 2 km from intervention streets, but this distance may not be sufficient to completely remove such effects. By using control streets in the same city, however, we could account for the many concurrent events or other interventions introduced during the same period that may have affected cycling behaviour while accounting for contextual influences.

### Strengths and limitations

Strengths include using a controlled ITS study design, which is among the most robust quasi-experimental study methods because it accounts for secular trends and other co-interventions or events [14]. We were also able to select from a wide range of potential control streets, allowing us to reduce possible confounding by matching similar streets in terms of pre-intervention trends and built environment features. In addition, by selecting other control streets in the sensitivity analysis, we could account for other potential sources of bias, such as whether the controls selected in the main analysis were representative of streets that did not receive interventions overall.

Some limitations include potential confounding by indication, whereby these cities may have chosen to either improve or extensively monitor those cycle lanes believed to have the largest likelihood of increasing cycling levels. Thus, it is unknown whether the cycle lanes we were able to evaluate (i.e., those with cycle counters installed before the intervention) were randomly selected or representative of other cycle lanes in each city. As the intervention allocation process is often opaque, natural experimental research cannot always avoid these problems. However, we took steps such as selecting control groups based on similar pre-intervention trends to reduce the risk of confounding.

There are also limitations to using cycle count data collected using automatic cycle counters, which allowed us to measure infrastructure usage but not necessarily cycling behaviour (e.g., frequency, duration) or whether certain population groups changed their cycling behaviour more than others. This is partly due to difficulties in ascertaining whether displacement occurred or when cyclists divert their route from nearby streets to improved intervention streets. However, collecting objective data to measure cycling behaviour often involves equipping individuals with accelerometer or GPS devices, which may be costly and requires intensive effort and resources. These measures are thus usually assessed in small samples and worn for a short period [7], the latter of which would preclude us from using ITS analysis. Nonetheless, the count data on infrastructural usage can support findings from other evaluations on cycling behaviour that measure outcomes more closely related to individual health impacts [7]. Moreover, ZELT cycle count technology has been found to undercount cyclists by 3% on separate paths and 4% on shared roadways [54], and the presence of bicycles with non-metallic (e.g., carbon fibre) wheels or groups of cyclists may also affect count data [32, 55]. However, any such undercounting is likely to have been small and equally applicable to all streets.

There are also challenges when using routinely collected data. For some streets, pre- and post-intervention periods could not be fully evaluated due to data availability. Although the effects of the study period length were examined in the sensitivity analysis for streets with the available data by extending the study period to one year before and after the intervention was implemented, we could not determine the long-term effects of these interventions. However, routinely collected data can offer additional opportunities in terms of what interventions can be evaluated and the methods used (e.g., ITS analysis). We were able to use routinely collected data to evaluate 15 streets, meaning this study is among the largest city-wide controlled natural experimental evaluations of new cycle lane infrastructure. Including interventions from two cities in France can also provide insights into how the effects of introducing new cycle infrastructure on cycling behaviour may differ according to existing relevant policies and local contexts. By taking wider contextual factors into account, we may in time be able to generalise such findings to other similar industrialised, high-density, and car-centric cities.

## Conclusions

The findings from this study suggest that improving or constructing new cycle lanes may be necessary but not sufficient to induce significant changes in cycling levels, as cycling levels increased in both Paris and Lyon across both intervention and control streets. This is likely due to these infrastructural changes forming only a part of a package of transportation interventions that were introduced in either city within the past decade. These have included interventions with carrot-and-stick strategies, or combinations of positive and negative motivators (e.g., implementing cycle lane improvements with congestion charging or removing parking spaces). Thus, cities should ensure that high-quality cycling infrastructure is available alongside other complementary interventions. Further research is needed to consider the incremental nature or lag effects of these interventions and increase understanding of the role of design and context on cycle lane effectiveness. As cities are turning towards urban design and transportation policies as solutions to wider environmental and health issues, this study demonstrates that a more comprehensive and system-wide approach may be needed to facilitate population shifts towards cycling.

## Supplementary Information

**Additional file 1.** **Appendix Table 1. **Intervention streets with selected control streets. **Appendix Table 2.** List of cycle lane infrastructure improvements and time before and after intervention was implemented Interrupted time series model. **Appendix Table 3.** Interrupted time series results for streets excluded from main analysis. **Appendix Table 4.** Interrupted time series and meta-analysis results for individual and city-wide pooled interventions. **Appendix Figure 1. **Interrupted time series analysis of selected streets in Paris with evidence of an impact due to a public transport strike. **Appendix Figure 2.** Forest plot of level change from interrupted time series sensitivity analysis results. **Appendix Figure 3.** Forest plot of trend change from interrupted time series sensitivity analysis results

## Data Availability

The dataset for Paris that was generated and analysed during the current study is available from Open Data Paris (https://opendata.paris.fr/explore/dataset/comptage-velo-donnees-compteurs/information/?disjunctive.id_compteur&disjunctive.nom_compteur&disjunctive.id&disjunctive.name). The dataset for Lyon is available upon reasonable request from the corresponding author.

## Abbreviations

ITS: Interrupted time series
Km: Kilometer
ARIMA: Autoregressive integrated moving average

## References

[1] World Health Organization. Urban health [Internet]. 2021 [cited 2021 Jul 23]. Available from: https://www.who.int/health-topics/urban-health

[2] United Nations Department of Economic and Social Affairs. World Urbanization Prospects: The 2018 Revision [Internet]. New York; 2019. Available from: https://population.un.org/wup/Publications/Files/WUP2018-Report.pdf

[3] Nieuwenhuijsen MJ. Urban and transport planning pathways to carbon neutral, liveable and healthy cities; A review of the current evidence. Environment International. Pergamon; 2020;140:105661. Available from: https://www.sciencedirect.com/science/article/pii/S0160412020302038 10.1016/j.envint.2020.105661 32307209

[4] Chapman R, Howden-Chapman P, Capon A. Understanding the systemic nature of cities to improve health and climate change mitigation. Environment International. Pergamon; 2016;94:380–7. Available from: https://www.sciencedirect.com/science/article/pii/S0160412016301441 10.1016/j.envint.2016.04.014 27126780

[5] Warburton DER, Nicol CW, Bredin SSD. Health benefits of physical activity: the evidence Review. Canadian Medical Association Journal. 2006;174:801. Available from: https://www.cmaj.ca/content/174/6/801#:~:text=In%20summary%2C%20observational%20studies%20provide,among%20asymptomatic%20men%20and%20women. 10.1503/cmaj.051351 PMC1402378 16534088

[6] World Health Organization. Physical activity [Internet]. 2018 [cited 2020 Jul 9]. Available from: https://www.who.int/news-room/fact-sheets/detail/physical-activity

[7] Mölenberg FJM, Panter J, Burdorf A, Van Lenthe FJ. A systematic review of the effect of infrastructural interventions to promote cycling: Strengthening causal inference from observational data. International Journal of Behavioral Nutrition and Physical Activity. BioMed Central Ltd.; 2019. p. 93. Available from: https://ijbnpa.biomedcentral.com/articles/10.1186/s12966-019-0850-1#:~:text=Most%20of%20the%20evaluations%20found,baseline%3A%2022%25%3B%20range%3A%20%E2%88%92 10.1186/s12966-019-0850-1 PMC6815350 31655609

[8] Stankov I, Garcia LMT, Mascolli MA, Montes F, Meisel JD, Gouveia N, et al. A systematic review of empirical and simulation studies evaluating the health impact of transportation interventions. Environmental Research. Academic Press; 2020;186:109519. Available from: https://www.sciencedirect.com/science/article/pii/S0013935120304126 10.1016/j.envres.2020.109519 PMC7343239 32335428

[9] Stappers NEH, van Kann DHH, Ettema D, de Vries NK, Kremers SPJ. The effect of infrastructural changes in the built environment on physical activity, active transportation and sedentary behavior – A systematic review. Health & Place. Pergamon; 2018;53:135–49. Available from: https://www.sciencedirect.com/science/article/pii/S1353829217311504?via%3Dihub 10.1016/j.healthplace.2018.08.002 30138827

[10] Mayne SL, Auchincloss AH, Michael YL. Impact of policy and built environment changes on obesity-related outcomes: A systematic review of naturally occurring experiments. Obesity Reviews [Internet]. Blackwell Publishing Ltd; 2015 [cited 2018 Nov 5];16:362–75. Available from: https://onlinelibrary.wiley.com/doi/full/10.1111/obr.12269 10.1111/obr.12269 PMC4789114 25753170

[11] Heesch KCKCKC, James B, Washington TLTLTL, Zuniga K, Burke M. Evaluation of the Veloway 1: A natural experiment of new bicycle infrastructure in Brisbane, Australia. Journal of Transport and Health [Internet]. Elsevier; 2016 [cited 2018 Nov 21];3:366–76. Available from: https://www.sciencedirect.com/science/article/pii/S2214140516301748

[12] Kontopantelis E, Doran T, Springate DA, Buchan I, Reeves D. Regression based quasi-experimental approach when randomisation is not an option: interrupted time series analysis. British Medical Journal [Internet]. British Medical Journal Publishing Group; 2015 [cited 2021 Jul 23];350. Available from: https://www.bmj.com/content/350/bmj.h2750 10.1136/bmj.h2750 PMC4460815 26058820

[13] Bernal JL, Cummins S, Gasparrini A. Interrupted time series regression for the evaluation of public health interventions: a tutorial. International Journal of Epidemiology [Internet]. Oxford Academic; 2017 [cited 2021 Jan 4];46:348–55. Available from: https://academic.oup.com/ije/article/46/1/348/2622842 10.1093/ije/dyw098 PMC5407170 27283160

[14] Bernal JL, Cummins S, Gasparrini A. The use of controls in interrupted time series studies of public health interventions. International Journal of Epidemiology [Internet]. Oxford Academic; 2018 [cited 2021 Aug 2];47:2082–93. Available from: https://academic.oup.com/ije/article/47/6/2082/5049576 10.1093/ije/dyy135 29982445

[15] Dennis J, Ramsay T, Turgeon AF, Zarychanski R. Helmet legislation and admissions to hospital for cycling related head injuries in Canadian provinces and territories: Interrupted time series analysis. British Medical Journal (Online) [Internet]. British Medical Journal Publishing Group; 2013 [cited 2021 Jul 15];346. Available from: https://pubmed.ncbi.nlm.nih.gov/23674137/10.1136/bmj.f2674 PMC3654159 23674137

[16] Liyanage P, Rocklöv J, Tissera H, Palihawadana P, Wilder-Smith A, Tozan Y. Evaluation of intensified dengue control measures with interrupted time series analysis in the Panadura Medical Officer of Health division in Sri Lanka: A case study and cost-effectiveness analysis. The Lancet Planetary Health. Elsevier; 2019;3:e211–8. Available from: https://www.thelancet.com/journals/lanplh/article/PIIS2542-5196(19)30057-9/fulltext 10.1016/S2542-5196(19)30057-9 31128766

[17] Thayer WM, Hasan MZ, Sankhla P, Gupta S. An interrupted time series analysis of the lockdown policies in India: a national-level analysis of COVID-19 incidence. Health Policy and Planning [Internet]. Oxford Academic; 2021 [cited 2021 Sep 8];36:620–9. Available from: https://academic.oup.com/heapol/article/36/5/620/6252392 10.1093/heapol/czab027 PMC8135431 33899097

[18] Turner S, Mackay D, Dick S, Semple S, Pell JP. Associations between a smoke-free homes intervention and childhood admissions to hospital in Scotland: An interrupted time-series analysis of whole-population data. The Lancet Public Health. Elsevier; 2020;5:e493–500. Available from: https://www.thelancet.com/journals/lanpub/article/PIIS2468-2667(20)30178-X/fulltext 10.1016/S2468-2667(20)30178-X 32888442

[19] Lee K, Sener IN. Emerging data for pedestrian and bicycle monitoring: Sources and applications. Transportation Research Interdisciplinary Perspectives. Elsevier; 2020;4:100095. Available from: https://www.sciencedirect.com/science/article/pii/S2590198220300063

[20] Prenveille C. Métropole de Lyon: Un net recul de la voiture [Internet]. Met’ Le magazine de la Métropole de Lyon. 2016 [cited 2021 Jul 23]. Available from: https://met.grandlyon.com/enquete-deplacements-recul-de-la-voiture/

[21] Fédération française des usagers de la bicyclette. Les villes qui aiment le vélo en France et à l’étranger [Internet]. [cited 2021 Jul 23]. Available from: https://www.fub.fr/velo-ville/villes-qui-aiment-velo/villes-qui-aiment-velo-france-etranger

[22] Ville de Paris. 2020: Paris, capitale du vélo, les objectifs pour la Ville [Internet]. 2021 [cited 2021 Jul 23]. Available from: https://www.paris.fr/pages/paris-a-velo-225

[23] Syndicat mixte des Transports pour le Rhône et l’Agglomération Lyonnaise. Le PDU 2017–2030 [Internet]. 2017 Nov. Available from: http://www.sytral.fr/306-presentation_pdu.htm

[24] Grand Lyon. 2012 - Plan d’actions partenarial [Internet]. Lyon; 2011 Nov. Available from: https://blogs.grandlyon.com/plan-climat/wp-content/blogs.dir/8/files/downloads/2011/12/Plan-daction-partenariat-BD.pdf

[25] Ville de Paris. Paris Respire [Internet]. 2021 [cited 2021 Jul 23]. Available from: https://www.paris.fr/pages/paris-respire-2122

[26] Paris sans voiture. Journée sans voiture [Internet]. 2021 [cited 2021 Jul 23]. Available from: https://www.parissansvoiture.org/

[27] Ville de Paris. La Zone à faibles émissions (ZFE) [Internet]. 2021 [cited 2021 Jul 23]. Available from: https://www.paris.fr/pages/la-zone-a-faibles-emissions-zfe-pour-lutter-contre-la-pollution-de-l-air-16799

[28] Ville de Paris. Paris Data [Internet]. 2021 [cited 2021 Jul 23]. Available from: https://opendata.paris.fr/pages/home/

[29] Grand Lyon. OnlyMoov [Internet]. 2021 [cited 2021 Sep 20]. Available from: https://data.eco-counter.com/ParcPublic/?id=3902

[30] Republique Francaise. Coronavirus: les mesures de confinement [Internet]. En bref. 2020 [cited 2020 Jun 26]. Available from: https://www.vie-publique.fr/en-bref/273932-coronavirus-les-mesures-de-confinement

[31] EcoCounter. Bike counters: how do they work? [Internet]. 2022 [cited 2022 Apr 12]. Available from: https://www.eco-counter.com/blog/bike-counters-how-do-they-work/

[32] Tin Tin S, Woodward A, Robinson E, Ameratunga S. Temporal, seasonal and weather effects on cycle volume: An ecological study. Environmental Health: A Global Access Science Source [Internet]. BioMed Central; 2012 [cited 2022 Apr 12];11:1–9. Available from: https://ehjournal.biomedcentral.com/articles/10.1186/1476-069X-11-12 10.1186/1476-069X-11-12 PMC3368741 22401535

[33] Pinheiro J, Bates D, DebRoy S, Sarkar D, R Core Team. {nlme}: Linear and Nonlinear Mixed Effects Models [Internet]. 2021. Available from: https://cran.r-project.org/package=nlme

[34] Bhaskaran K, Gasparrini A, Hajat S, Smeeth L, Armstrong B. Time Series Regression Studies in Environmental Epidemiology. International Journal of Epidemiology. 2013;42:1187–95. Available from: https://www.ncbi.nlm.nih.gov/pmc/articles/PMC3780998/10.1093/ije/dyt092 PMC3780998 23760528

[35] Peng RD, McDermott A. tsModel: Time Series Modeling for Air Pollution and Health [Internet]. 2013. Available from: https://cran.r-project.org/package=tsModel

[36] Dolbois M. Grève à la RATP et à la SNCF lundi 27 janvier: trafic normal sur toutes les lignes de métro, RER, Transilien. Actu Seine-Saint-Denis [Internet]. Paris; 2020 Jan 20 [cited 2021 Jul 23]; Available from: https://actu.fr/societe/greve-la-ratp-a-sncf-lundi-27-janvier-trafic-normal-sur-toutes-lignes-metro-rer-transilien_31034479.html

[37] Fuller D, Sahlqvist S, Cummins S, Ogilvie D (2012). The impact of public transportation strikes on use of a bicycle share program in London: Interrupted time series design. Preventive Medicine Academic Press.

[38] Fuller D, Luan H, Buote R, Auchincloss AH (2019). Impact of a public transit strike on public bicycle share use: An interrupted time series natural experiment study. Journal of Transport and Health Elsevier.

[39] Balduzzi S, Rücker G, Schwarzer G (2019). How to perform a meta-analysis with {R}: A practical tutorial. Evid Based Ment Health.

[40] R Core Team (2020). R: A language and environment for statistical computing.

[41] Halpern SD, French B, Small DS, Saulsgiver K, Harhay MO, Audrain-McGovern J, et al. Randomized Trial of Four Financial-Incentive Programs for Smoking Cessation. New England Journal of Medicine [Internet]. Massachusetts Medical Society; 2015 [cited 2021 Mar 30];372:2108–17. Available from: http://www.nejm.org/doi/10.1056/NEJMoa1414293 10.1056/NEJMoa1414293 PMC4471993 25970009

[42] Petrunoff N, Rissel C, Wen LM, Martin J. Carrots and sticks vs carrots: Comparing approaches to workplace travel plans using disincentives for driving and incentives for active travel. Journal of Transport and Health. Elsevier Ltd; 2015;2:563–7. Available from: https://www.sciencedirect.com/science/article/pii/S2214140515006544?via%3Dihub

[43] Aldred R, Elliott B, Woodcock J, Goodman A. Cycling provision separated from motor traffic: A systematic review exploring whether stated preferences vary by gender and age. Transport Reviews [Internet]. Routledge; 2016 [cited 2021 Aug 2];37:29–55. Available from: https://www.tandfonline.com/doi/abs/10.1080/01441647.2016.1200156 10.1080/01441647.2016.1200156 PMC5259802 28190905

[44] Garrard J, Rose G, Lo SK. Promoting transportation cycling for women: The role of bicycle infrastructure. Preventive Medicine. Academic Press; 2008;46:55–9. Available from: https://www.sciencedirect.com/science/article/pii/S0091743507003039 10.1016/j.ypmed.2007.07.010 17698185

[45] Skov-Petersen H, Jacobsen JB, Vedel SE, Thomas Alexander SN, Rask S. Effects of upgrading to cycle highways - An analysis of demand induction, use patterns and satisfaction before and after. Journal of Transport Geography. Pergamon; 2017;64:203–10. Available from: https://www.sciencedirect.com/science/article/pii/S0966692316304008

[46] Fitzhugh EC, Bassett DR, Evans MF. Urban trails and physical activity: A natural experiment. American Journal of Preventive Medicine [Internet]. Am J Prev Med; 2010 [cited 2021 Mar 8];39:259–62. Available from: https://pubmed.ncbi.nlm.nih.gov/20709258/10.1016/j.amepre.2010.05.010 20709258

[47] Panter J, Heinen E, Mackett R, Ogilvie D. Impact of New Transport Infrastructure on Walking, Cycling, and Physical Activity. American Journal of Preventive Medicine [Internet]. Elsevier; 2016 [cited 2018 Jul 24];50:e45–53. Available from: https://pubmed.ncbi.nlm.nih.gov/26585051/10.1016/j.amepre.2015.09.021 PMC4712020 26585051

[48] Dill J, McNeil N, Broach J, Ma L. Bicycle boulevards and changes in physical activity and active transportation: Findings from a natural experiment. Preventive Medicine. Academic Press; 2014;69:S74–8. Available from: https://www.sciencedirect.com/science/article/pii/S0091743514003703?via%3Dihub 10.1016/j.ypmed.2014.10.006 25456802

[49] Crane M, Rissel C, Standen C, Ellison A, Ellison R, Wen LM, et al. Longitudinal evaluation of travel and health outcomes in relation to new bicycle infrastructure, Sydney, Australia. Journal of Transport and Health. Elsevier Ltd; 2017;6:386–95. Available from: https://www.sciencedirect.com/science/article/pii/S2214140517301500?via%3Dihub

[50] Goodman A, Sahlqvist S, Ogilvie D. New walking and cycling routes and increased physical activity: One- and 2-year findings from the UK iConnect study. American Journal of Public Health [Internet]. American Public Health Association Inc.; 2014 [cited 2021 Mar 8];104:e38. Available from: https://www.ncbi.nlm.nih.gov/pmc/articles/PMC4151955/10.2105/AJPH.2014.302059 PMC4151955 25033133

[51] Taljaard M, McKenzie JE, Ramsay CR, Grimshaw JM. The use of segmented regression in analysing interrupted time series studies: An example in pre-hospital ambulance care. Implementation Science 2014 9:1 [Internet]. BioMed Central; 2014 [cited 2021 Aug 2];9:1–4. Available from: https://implementationscience.biomedcentral.com/articles/10.1186/1748-5908-9-77 10.1186/1748-5908-9-77 PMC4068621 24943919

[52] APUR. Évolution des mobilités dans le Grand Paris Tendances historiques, évolutions en cours et émergentes [Internet]. 2021 [cited 2022 Apr 12]. Available from: https://www.apur.org/fr/nos-travaux/evolution-mobilites-grand-paris-tendances-historiques-evolutions-cours-emergentes

[53] Urbalyon. Vélo: Evolutions des pratiques et potentiels de développement [Internet]. 2021 [cited 2022 Apr 12]. Available from: https://www.urbalyon.org/fr/observatoire-des-deplacements/velo-evolutions-des-pratiques-et-potentiels-de-developpement

[54] Nordback K, Piatkowski D, Janson BN, Marshall W, Krizek KJ, Main D. Testing Inductive-Loop Bicycle Counters on Shared Roadways. Transportation Research Board 90th Annual Meeting [Internet]. Washington DC; 2011 [cited 2022 Apr 12]. Available from: https://trid.trb.org/view/1093482

[55] ViaStrada Ltd, New Zealand Transport Agency. Continuous cycle counting trial [Internet]. Christchurch; 2009. Available from: https://www.nzta.govt.nz/assets/resources/continuous-cycle-counting-trial/docs/continuous-cycle-counting-trial.pdf

