# Supplementary material for: Impacts of new cycle infrastructure on cycling levels in two French cities: an interrupted time series analysis
Source: Int J Behav Nutr Phys Act. 2022 Jul 7;19:77. doi: 10.1186/s12966-022-01313-0 (PMC9260999; doi:10.1186/s12966-022-01313-0)

Appendix

**Appendix Table 1.** Intervention streets with selected control streets

**Appendix Table 2.** List of cycle lane infrastructure improvements and time before and after intervention was implemented

**Interrupted time series model**

**Appendix Table 3.** Interrupted time series results for streets excluded from main analysis

**Appendix Figure 1.** Interrupted time series analysis of selected streets in Paris with evidence of an impact due to a public transport strike

**Appendix Table 4.** Interrupted time series and meta-analysis results for individual and city-wide pooled interventions

**Appendix Figure 2.** Forest plot of level change from interrupted time series sensitivity analysis results

**Appendix Figure 3.** Forest plot of trend change from interrupted time series sensitivity analysis results

**Appendix Table 1.** Intervention streets with selected control streets

|  | Intervention street | Control street |
| --- | --- | --- |
| **Paris** |  | |
| A | Rue de Rivoli | Boulevard de Magenta |
| B | Boulevard Voltaire | Rue la Fayette |
| C | Rue Julia Bartet | Rue la Fayette |
| D | Boulevard Diderot | Avenue Denfert Rochereau |
| E | Rue d'Aubervilliers | Boulevard Auguste Blanqui |
| F | Avenue de la Porte des Ternes | Quai de l'Hotel de ville |
| G | Rue de Turbigo | Avenue Denfert Rochereau |
| H | Rue Lecourbe | Boulevard Auguste Blanqui |
| **Lyon** |  | |
| I | Rue Victor Lagrange | Quai de Pierre-Scize |
| J | Quai Jaÿr | Rue Paul Duvivier |
| K | Cours Gambetta | Quai de Pierre-Scize |
| L | Quai Claude Bernard | Pont de la Feuillée |
| M | Rue de la Viabert | Rue Raoul Servant |
| N | Cours Albert Thomas | Place Bellecour |
| O | Avenue Félix Faure | Avenue Jean Jaurès |
| P | Rue Vauban | Rue Raoul Servant |
| Q | Rue Rabelais | Rue Raoul Servant |
| R | Boulevard Pinel | Boulevard des États-Unis |

**Appendix Table 2.** List of cycle lane infrastructure improvements and time before and after intervention was implemented

| Street Name | Cycling counter Built | Infrastructure delivered | Before data (months) | After data (months) | Total data (months) |
| --- | --- | --- | --- | --- | --- |
| Paris |  |  |  |  |  |
| Rue de Rivoli | 30/09/2008 | 18/09/2019 | 131.0 | 6.0 | 137.0 |
| Boulevard Diderot | 17/01/2013 | 31/05/2019 | 77.5 | 9.7 | 87.2 |
| Boulevard Voltaire | 26/06/2018 | 31/08/2019 | 14.4 | 6.7 | 21.0 |
| Rue Julia Bartet | 27/11/2018 | 20/12/2019 | 12.9 | 3.0 | 15.9 |
| Rue d'Aubervilliers | 28/11/2018 | 30/10/2019 | 11.2 | 4.7 | 15.9 |
| Rue de Turbigo | 31/10/2019 | 31/12/2019 | 2.0 | 2.7 | 4.7 |
| Avenue de la Porte des ternes | 28/11/2018 | 31/12/2018 | 1.1 | 14.9 | 16.0 |
| Rue Lecourbe | 16/10/2019 | 06/11/2019 | 0.7 | 4.6 | 5.3 |
| Lyon |  |  |  |  |  |
| Cours Gambetta | 09/07/2008 | 25/08/2015 | 86.8 | 55.8 | 142.5 |
| Rue de la Viabert | 16/04/2012 | 02/10/2014 | 30.0 | 66.7 | 96.7 |
| Rue Vauban | 16/04/2012 | 31/03/2019 | 84.7 | 12.0 | 96.7 |
| Quai Claude Bernard | 03/07/2016 | 31/12/2018 | 30.4 | 15.1 | 45.4 |
| Rue Rabelais | 18/11/2013 | 19/07/2018 | 56.8 | 20.6 | 77.4 |
| Cours Albert Thomas | 07/12/2015 | 25/05/2018 | 30.0 | 22.5 | 52.5 |
| avenue Felix Faure | 13/04/2011 | 25/05/2018 | 86.6 | 22.5 | 109.1 |
| Quai Jayr | 24/06/2014 | 31/03/2018 | 45.9 | 24.4 | 70.2 |
| Boulevard Pinel | 01/02/2018 | 02/09/2019 | 19.3 | 7.1 | 26.3 |
| Rue Victor Lagrange | 25/10/2012 | 03/12/2019 | 86.5 | 4.0 | 90.5 |

**Interrupted time series model**

To choose the most appropriate regression model, a generalized linear model was compared against a Poisson and negative binomial regression model, which are commonly used for count data. The generalized linear model was considered the most appropriate as it had the best fit after calculating log-likelihood and AIC scores. Seasonality was modelled using Fourier terms with four sine and cosine pairs over twelve months and autocorrelation was assessed using autocorrelation (ACF) and partial autocorrelation plots (PACF). As the model was determined to be an autoregressive process, it was therefore adjusted with a lag of p = 7; further model-fitting tests such as log-likelihood and AIC test revealed that adjusting for a p = 7 and q = 2 lag was more appropriate.

$$Y_{i}= {\alpha+ \beta_{1}T}_{i}+{\beta_{2}X}_{i} +{\beta_{3}X}_{i}T+{\beta_{4}Z}_{i}+{\beta_{5}Z}_{i}T+{\beta_{6}Z}_{i}X_{i}+{\beta_{7}Z}_{i}X_{i}T+ \varepsilon_{i}$$

α = expected cycle count in the control street in the pre-intervention period

$\beta_{1}$ = control street trend in the pre-intervention period

$\beta_{2}$ = difference in level between post-intervention and pre-intervention periods in the control street

$\beta_{3}$ = difference in trend between post-intervention and pre-intervention periods in the control street

$\beta_{4}$ = difference in level between intervention and control street in the pre-intervention period

$\beta_{5}$ = difference in trend between intervention and control street in the pre-intervention period

$\beta_{6}$ = difference in level between intervention and control street in the post-intervention period

$\beta_{7}$ = difference in trend between intervention and control street in the post-intervention period

$T$ = time elapsed since the start of the study in days

$Z$ = dummy variable indicating the intervention group (1 = treatment, 0 = control)

$i$ = dummy variable indicating the period (0 = pre-intervention, 1 = post-intervention)

$Y_{i}$ = the outcome at time i

**Appendix Table 3.** Interrupted time series results for streets excluded from main analysis

|  | **Level Change** | | | | **Trend Change** | | | |
| --- | --- | --- | --- | --- | --- | --- | --- | --- |
| Intervention | Value | LCI | UCI | p-value | Value | LCI | UCI | p-value |
| **Paris** |  |  |  |  |  |  |  |  |
| Avenue de la Porte des Ternes | 19.95 | -346.99 | 386.9 | 0.92 | -11.95 | -30.68 | 6.78 | 0.21 |
| Rue de Turbigo | 921 | -379.37 | 2220.94 | 0.17 | -14 | -43.18 | 15.52 | 0.36 |
| Rue Lecourbe | -26.4 | -944.03 | 891.28 | 0.96 | -79.6 | -306.34 | 147.1 | 0.49 |

* LCI: Lower confidence interval; UCI: Upper confidence interval

**Appendix Figure 1.** Interrupted time series analysis of select streets in Paris with evidence of an impact due to a public transport strike


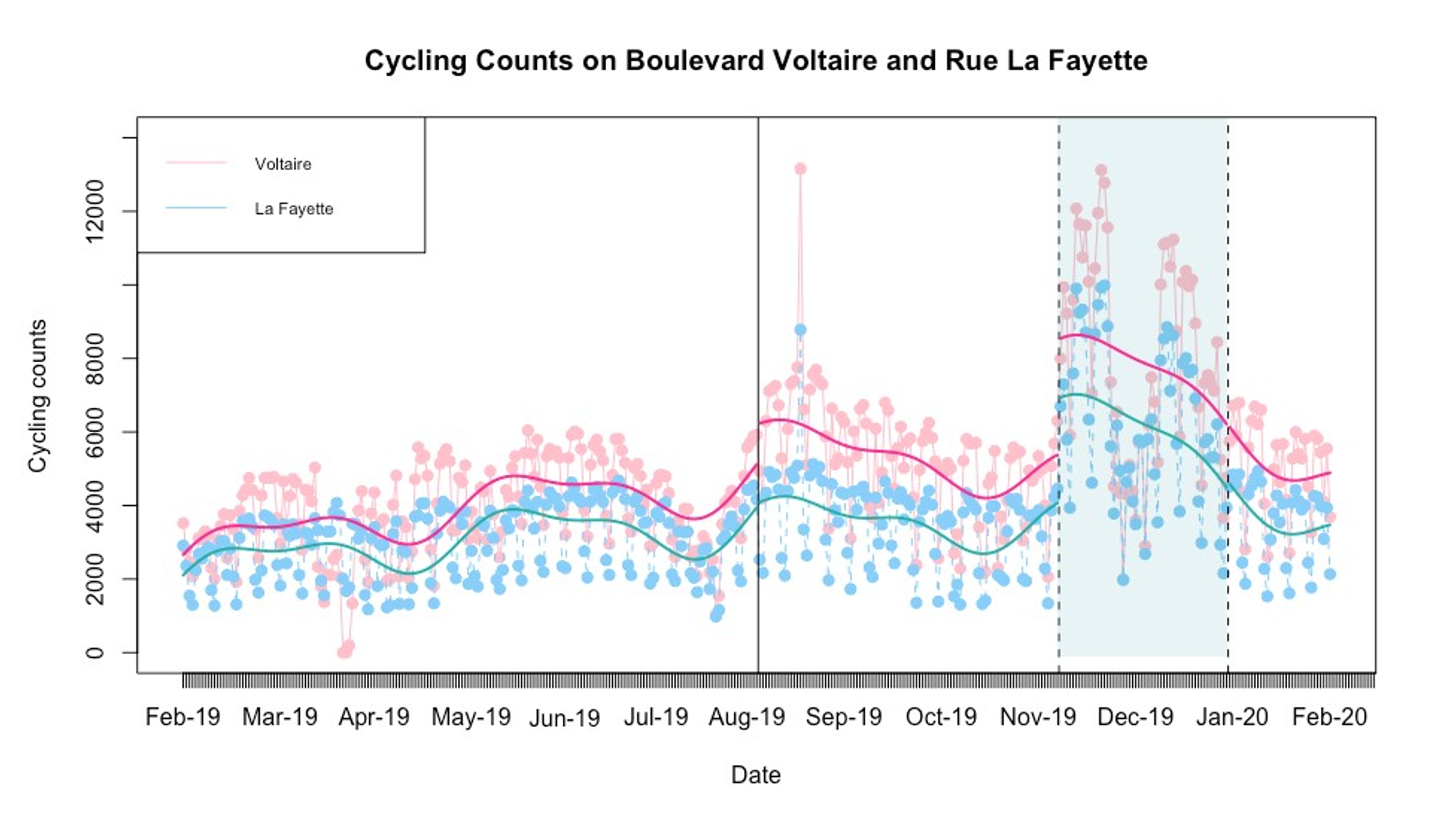


Note: The solid vertical line denotes the date the intervention was implemented; dashed lines represent the start and end of the strike, also shaded in blue. Counts are in daily cycle counts.

**Appendix Table 4.** Interrupted time series and meta-analysis results for individual and city-wide pooled interventions

|  | **Level change** | | | | **Trend change** | | | |
| --- | --- | --- | --- | --- | --- | --- | --- | --- |
| Intervention | Value | LCI | UCI | p-value | Value | LCI | UCI | p-value |
| **Paris** | | | | | | | | |
| Rivoli | 619.03 | -1700.46 | 2938.53 | 0.6 | -16.59 | -60.7 | 27.52 | 0.46 |
| Voltaire | 982.19 | -627.05 | 2591.44 | 0.23 | -12.11 | -43.05 | 18.84 | 0.44 |
| Julia Bartet | 799.36 | -539.05 | 2137.78 | 0.24 | 168.15 | 10.97 | 325.32 | 0.04 |
| Diderot | 199.97 | -288.46 | 688.41 | 0.42 | -0.18 | -6.4 | 6.05 | 0.96 |
| Aubervilliers | -36.15 | -439.97 | 367.66 | 0.86 | -3.95 | -22.93 | 15.02 | 0.68 |
| **Lyon** | | | | | | | | |
| Victor Lagrange | 483.12 | -74.19 | 1040.44 | 0.09 | -7.94 | -17.7 | 1.83 | 0.11 |
| Jayr | 154.23 | -94.82 | 346.5 | 0.16 | 1.17 | -1.72 | 4.06 | 0.43 |
| Gambetta | 284.73 | -282 | 851.47 | 0.33 | -7.64 | -13.61 | -1.66 | 0.01 |
| Claude Bernard | -77.09 | -667.89 | 513.71 | 0.8 | 2.53 | -4.2 | 9.26 | 0.46 |
| Viabert | 81.99 | -59.63 | 223.61 | 0.26 | -0.78 | -2.19 | 0.63 | 0.28 |
| Albert Thomas | -71.67 | -839.21 | 695.87 | 0.85 | 1.69 | -8.49 | 11.88 | 0.74 |
| Felix Faure | -97.71 | -549.93 | 354.51 | 0.67 | -1.29 | -7.51 | 4.93 | 0.69 |
| Vauban | 2.01 | -284.29 | 288.3 | 0.99 | -0.82 | -4.33 | 2.69 | 0.65 |
| Rabelais | -34.02 | -241.49 | 173.45 | 0.75 | -0.02 | -2.48 | 2.45 | 0.99 |
| Pinel | -71.66 | -148.61 | 5.29 | 0.07 | 0.42 | -0.38 | 1.23 | 0.3 |
| **Meta-analysis** | | | | | | | | |
| Paris Pooled | 218.01 | -189.57 | 625.58 | 0.21 | 7.02 | -59.18 | 73.22 | 0.78 |
| Lyon Pooled | 33.71 | -65.22 | 132.63 | 0.46 | 0.03 | -2.68 | 1.28 | 0.44 |
| Paris and Lyon Pooled | 68.1 | -35.92 | 172.11 | 0.18 | -1.25 | -9 | 6.41 | 0.72 |

* LCI: Lower confidence interval; UCI: Upper confidence interval

**Appendix Figure 2.** Forest plot of level change from interrupted time series sensitivity analysis results


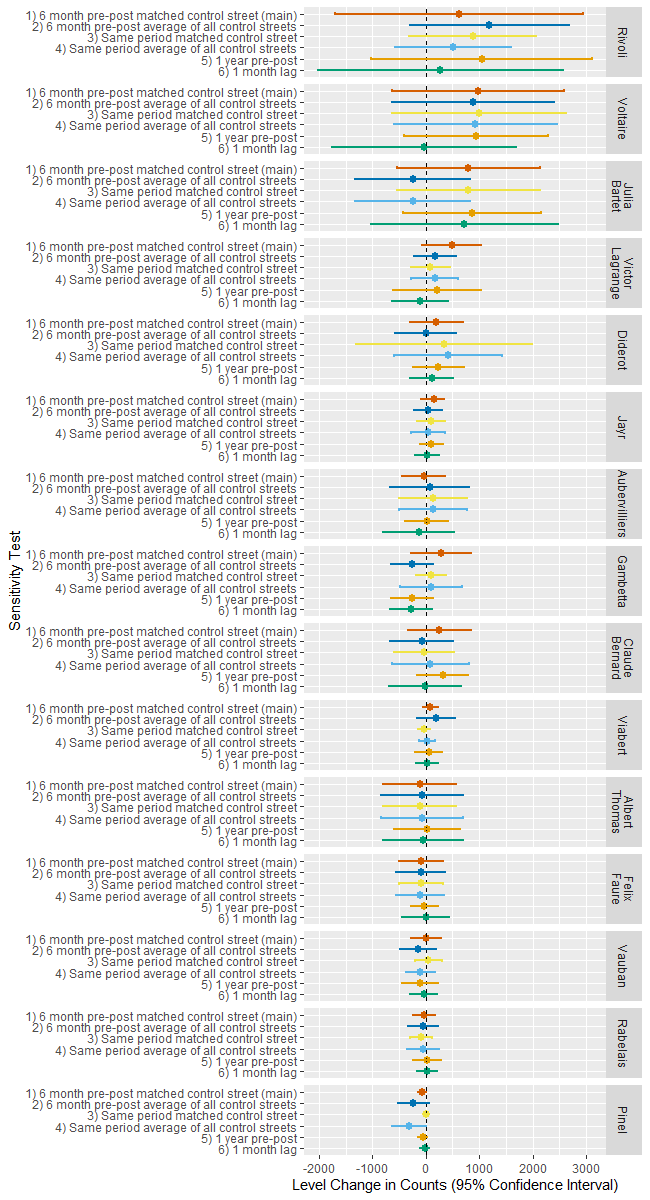


**Appendix Figure 3.** Forest plot of trend change from interrupted time series sensitivity analysis results


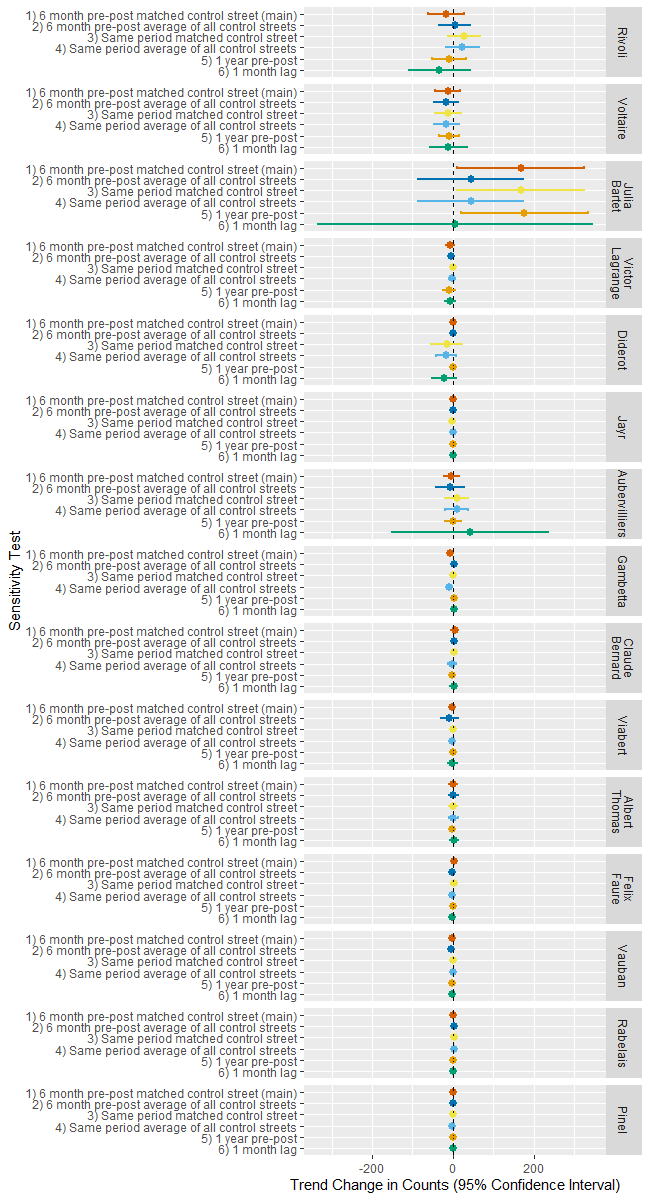

Supplement: Supplementary file 1 — Additional file 1. Appendix Table 1. Intervention streets with selected control streets. Appendix Table 2. List of cycle lane infrastructure improvements and time before and after intervention was implemented Interrupted time series model. Appendix Table 3. Interrupted time series results for streets excluded from main analysis. Appendix Table 4. Interrupted time series and meta-analysis results for individual and city-wide pooled interventions. Appendix Figure 1. Interrupted time series analysis of selected streets in Paris with evidence of an impact due to a public transport strike. Appendix Figure 2. Forest plot of level change from interrupted time series sensitivity analysis results. Appendix Figure 3. Forest plot of trend change from interrupted time series sensitivity analysis results [file 12966_2022_1313_MOESM1_ESM.docx]
